# Soil Respiration of the Dahurian Larch (*Larix gmelinii*) Forest and the Response to Fire Disturbance in Da Xing’an Mountains, China

**DOI:** 10.1038/s41598-017-03325-4

**Published:** 2017-06-07

**Authors:** Tongxin Hu, Long Sun, Haiqing Hu, David R. Weise, Futao Guo

**Affiliations:** 1 College of Forestry, Northeast Forestry University, Harbin, 150040 China; 2 USDA Forest Service, PSW Research Station, 4955 Canyon Crest Drive, Riverside, CA 92507 USA; 3 College of Forestry, Fujian Agriculture and Forestry University, Fuzhou, 350002 China

## Abstract

Despite the high frequency of wildfire disturbances in boreal forests in China, the effects of wildfires on soil respiration are not yet well understood. We examined the effects of fire severity on the soil respiration rate (*R*s) and its component change in a Dahurian Larch (*Larix gmelinii*) in Northeast China. The results showed that *R*s decreased with fire burning severity. Compared with the control plots, *R*s in the low burning severity plots decreased by 19%, while it decreased by 28% in the high burning severity plots. The *R*s decrease was mainly due to a decreased autotrophic respiration rate (*R*a). The temperature sensitivity (*Q*_10_) of *R*s increased after the low severity fire disturbances, but it decreased after the high severity fire disturbance. The *R*s were triggered by the soil temperature, which may explain most of the *R*s variability in this area. Our study, for the first time, provides the data-based foundation to demonstrate the importance of assessing CO_2_ fluxes considering both fire severity and environmental factors post-fire in boreal forests of China.

## Introduction

The soil respiration rate (*R*s) is the second largest carbon flux (80–98 Pg C·yr^−1^) in terrestrial ecosystems^1^. Soil respiration is the sum of soil autotrophic respiration (*R*a) from plant metabolic activity and soil heterotrophic respiration (*R*h) from the decomposition of organic material by microbes^2^. The amount of CO_2_ released by soil respiration is more than ten times that released by global fossil fuel combustion^3^; Slight changes in soil respiration may therefore influence the global carbon balance^4^. Understanding the mechanisms and potential changes of soil and CO_2_ exchange as a function of soil respiration is key to learning about the forest ecosystem response to global climate change^2^.

Recent soil respiration research has focused on the effects of disturbances on soil respiration, such as prescribed burning, litter thinning, harvesting removal, nitrogen addition and land use management^5, 6^. However, despite the global importance of this process, there is still a lack of understanding of the variability of soil respiration in high-latitude boreal ecosystems^7^. The boreal forests of Asia, Europe, and North America contain approximately 40% of the global soil organic carbon, roughly the same value as atmospheric carbon, making the global boreal ecosystem the largest terrestrial organic carbon pool^8^. Boreal forest carbon sequestration and emission is largely determined by forest fire disturbances^9^, and the frequent and severe forest fires significantly affect the carbon balance in these ecosystems^10^. The fire return interval and fire severity have increased significantly over the past few decades^11^. The carbon loss in boreal forest soil caused by fire disturbance is not only an important factor in determining forest carbon balance but also a point of uncertainty in global carbon assessment^12^. Much of this uncertainty stems from the high degree of soil heterogeneity^13, 14^, as well as the complex interactions between differences in the soil environment characteristics and forest fire^15^. The fire duration and severity and the meteorological condition post-fire can also significantly influence soil respiration after fire disturbance, which can last a few months to a few years^16^. Therefore, understanding the regime of soil respiration and its affecting factors after fire disturbance can enhance the accuracy of estimating soil respiration in boreal forest ecosystems.

Fire can increase soil hydrophobicity, which may indirectly control the rates of decomposition by reducing the soil moisture infiltration and increasing the surface runoff^17^. Wildfires can also affect soil respiration by reducing vegetation cover and surface albedo, which increases soil temperatures and decomposition rates^5, 18^. Forest fires can oxidize part to all forest vegetation, which in turn affects soil temperature, moisture, microbial activity, and root composition, and then significantly affects soil respiration^19^. Additionally, the *Q*_10_ value, the factor by which soil respiration is multiplied when the temperature increases by 10 degrees, also varies with forest fire severity^20^. Generally, the *Q*_10_ increases with decreasing temperature and increasing moisture on large scales and is also dependent on the substrate quality and availability^21^. The quantity and quality of detritus and root on the ground and underground varies with the duration and severity of forest fires^22, 23^. Several studies^18, 24, 25^ have explored the effects of fire disturbance on the component of soil respiration (microbial and root respiration), but there is much uncertainty that requires further study. The previous results indicated that the quantitative relationship between soil temperature moisture and the change in soil respiration components is the key to understanding the response of forest ecosystems to fire disturbance^13^.

The Daxing’an Mountains are the largest area of boreal forests in China. The dominant vegetation is the Dahurian Larch (*Larix gmelinii*) forest, which accounts for 70% of the total forest area in the Daxing’an Mountains^26^. It is the southern edge of the Eurasian boreal forest, a cold-temperate forest transition zone, which is very sensitive to rapid climate changes^27^. The Daxing’an Mountains have the highest incidence of forest fires in China. In total, 1,614 fires occurred from 1965 to 2010. The effect of fire disturbance on the carbon cycle in a Dahurian Larch (*Larix gmelinii*) forest will help to elucidate the role played by the boreal forest of China in the process of global carbon balance. However, the Dahurian Larch (*Larix gmelinii*) forest of China has received only limited attention, and there is much uncertainty about soil respiration following fire disturbance in the background of global climate change.

This study aims to (1) quantify the soil autotrophic and heterotrophic respiration of the Dahurian Larch (*Larix gmelinii*) forest during the growing season, (2) compare the effects of different severities of forest fire on soil respiration, and (3) identify the factors influencing soil respiration changes after fires.

## Results

### Effects of fire disturbance on soil respiration and environmental factors

**Figure 1:**
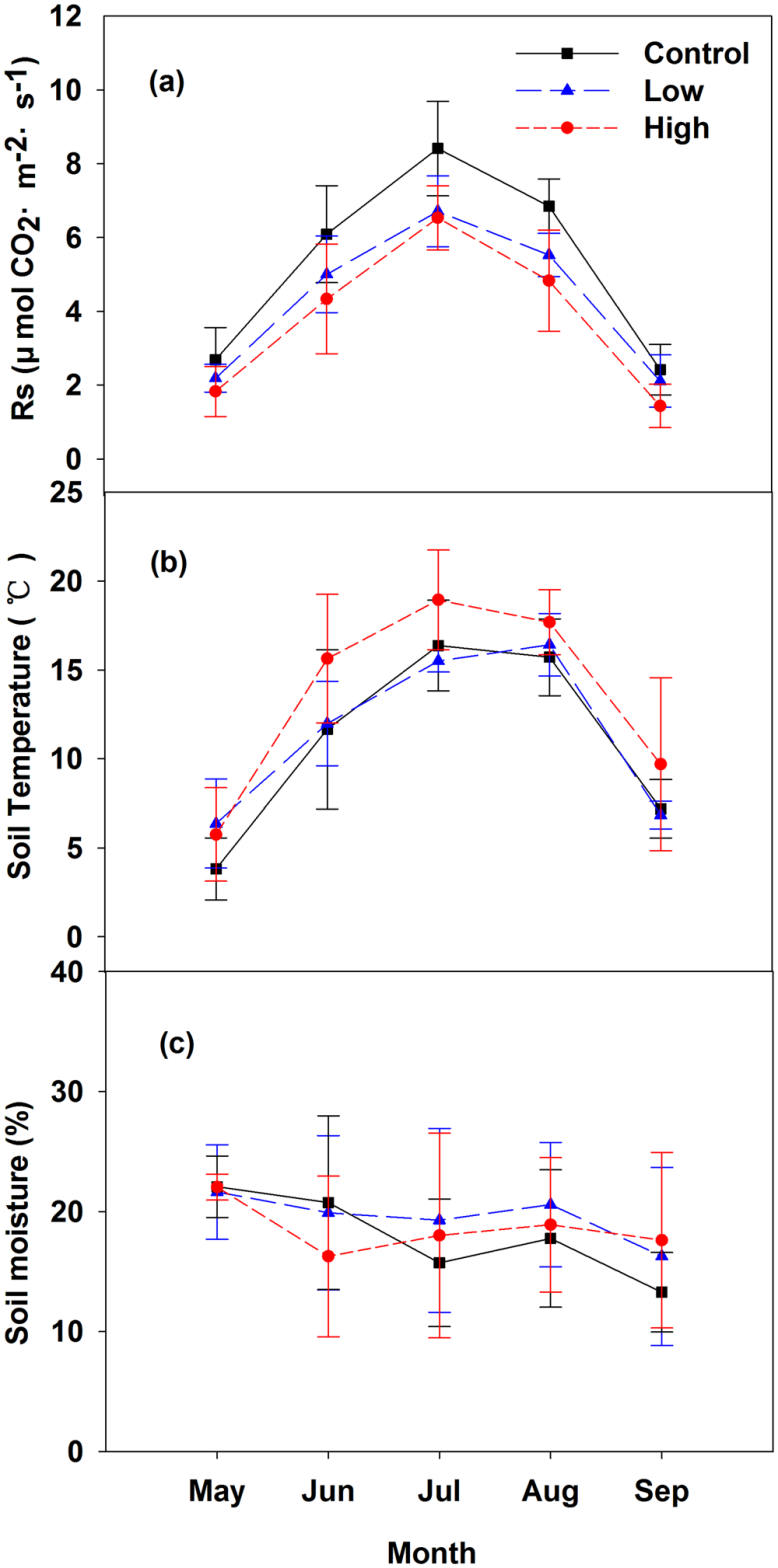
Seasonal variations of (**a**) the total soil respiration rates (*R*s), (**b**) soil temperature and (**c**) soil moisture in the control, low, and high burning severity plots. The data were the average values in 2010, 2011, and 2012. The error bars represent the standard deviation.

Statistically significant seasonal variations of *R*s were observed in all three types of plots (control, low, and high burning severity) during the growing season (*P* < 0.05), and the overall trend of *R*s increased from early May to late July and then decreased until late September. The seasonal trend of *R*s in all three types of plots showed a single peak curve (Fig. 1a). The mean values of *R*s in the control, low, and high burning severity plots was 5.29 ± 0.48, 4.31 ± 0.36, and 3.79 ± 0.25 μmol CO_2_·m^−2^ s^−1^, respectively. Compared with the control plots, the average *R*s in the low and high burning severity plots decreased by approximately 19% and 28%, respectively. The *R*s in the high burning severity plot was significantly lower than that in the control plot (*P* < 0.05).

The maximum soil temperature occurred in July, while the minimum value was obtained in May. The seasonal dynamic variations of *T* were significantly observed in all three types of plots (Fig. 1b) (*P* < 0.05). The mean soil temperatures in the control, low, and high burning severity plots were 10.95 ± 1.43, 11.42 ± 1.2, and 13.54 ± 1.8 °C, respectively. There was no significant difference in the seasonal dynamic of soil moisture in the three types of plots (*P* > 0.05) (Fig. 1c). The mean soil moisture in the control, low, and high burning severity plots was 18% ± 3%, 20% ± 5%, and 19% ± 2%, respectively.

The seasonal variation of *R*s was closely related to the soil temperature changes at a 5-cm depth rather than to the soil moisture. The change in *R*s was consistent with soil temperature, whereas soil moisture did not show a close relationship with *R*s (Fig. 1).

### Effects of fire disturbance on soil respiration components

**Figure 2:**
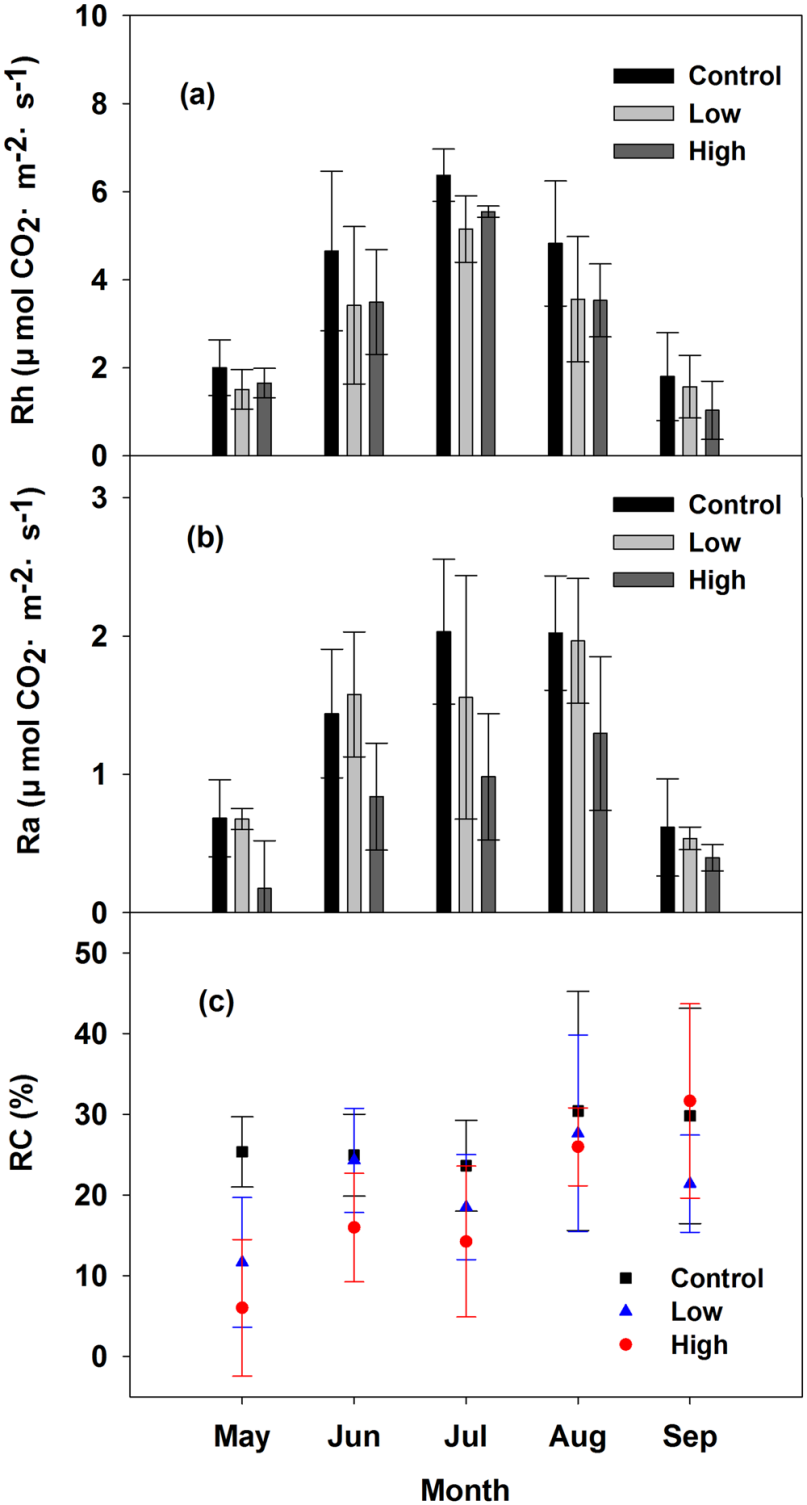
Seasonal variations of (**a**) the soil heterotrophic respiration rates (*R*h), (**b**) the soil autotrophic respiration rates (*R*a) and (**c**) the RC (*R*a:*R*s) in the control, low, and high burning severity plots. The data were the average values in 2010, 2011, 2012. The error bars represent the standard deviation.

Seasonal patterns of *R*h and *R*a were similar to that of *R*s (Fig. 2a,b). Both *R*h and *R*a increased from early May to late July. The annual mean *R*h in the control, low, and high burning severity plots was 3.93 ± 0.71, 3.04 ± 0.81, and 3.05 ± 0.26 μmol CO_2_·m^−2^ s^−1^, respectively. No significant difference between the *R*h in the control plot and that in the high and low burning severity plots (*P* > 0.05) was found. The annual mean *R*a in the control, low, and high burning severity plots was 1.36 ± 0.24, 1.26 ± 0.48, 0.74 ± 0.31 μmol CO_2_·m^−2^ s^−1^, respectively. Compared with the control and low burning severity plots, the annual mean *R*a in the high burning severity plot significantly decreased by approximately 46% and 41%, respectively. The average RC (*R*a:*R*h) in the control, low, and high burning severity plots was approximately 27%, 29%, and 19%, respectively (Fig. 2c).

### Relationships between soil respiration and environmental factors

The exponential regression model with soil temperature as a single controlling factor of *R*s and *R*h were significant for the control, low and high burning severity plots and explained approximately 50~60% and 34%~60% of variation in *R*s and *R*h, respectively (Table 1). The models that fit soil temperature, soil moisture, and their interaction could explain approximately 50~67% and 43~60% of the variability in *R*s and *R*h, respectively, in different fire burning severity plots (Table 1). Although soil temperature and soil moisture together can improve the correlation coefficients (*R* ^2^) of the *R*s and *R*h regression models in all types of plots, the soil temperature is still the dominant factor controlling the variability of *R*s and *R*h in this region.

**Table 1:** Summary of the regression models of soil respiration against soil temperature (*L*n(*R*s) = *α* + *β* × *T*) and the best-fit regression models of soil respiration against soil temperature and soil moisture (*L*n(*R*s) = *α* + *β* × *T* + *ε* × *W* + *ω* × *T* × *W*), where *T* is the soil temperature (°C), *W* is the soil moisture (%), and *T* × *W* is the interaction effect of *T* and *W*.

Site	Respiration	*α*	*β*	*ε*	*ω*	*Q*_10_	*R* ^2^
Control	*R*s	0.569 ± 0.059	0.086 ± 0.005	b	b	2.36	0.6
	*R*s	0.164 ± 0.082	0.094 ± 0.005	1.747 ± 0.266	a		0.67
	*R*h	−0.143 ± 0.1	0.109 ± 0.008	b	b	2.97	0.6
	*R*h	−0.143 ± 0.1	0.109 ± 0.008	a	a		0.6
Low	*R*s	−0.025 ± 0.073	0.104 ± 0.006	b	b	2.83	0.59
	*R*s	−0.025 ± 0.073	0.104 ± 0.006	a	a		0.59
	*R*h	−0.310 ± 0.09	0.10 ± 0.007	b	b	2.72	0.55
	*R*h	−0.337 ± 0.89	0.082 ± 0.009	a	0.095 ± 0.031		0.57
High	*R*s	−0.060 ± 0.086	0.077 ± 0.006	b	b	2.01	0.5
	*R*s	−0.063 ± 0.083	0.061 ± 0.006	a	0.145 ± 0.026		0.52
	*R*h	−0.109 ± 0.112	0.069 ± 0.007	b	b	1.99	0.34
	*R*h	−0.839 ± 0.173	0.081 ± 0.007	2.726 ± 0.516	a		0.43

Note ^a^that this variable of the model was not significant in an ANOVA (at the *P* = 0.05 level), ^b^and this variable was not included in the model.

**Figure 3:**
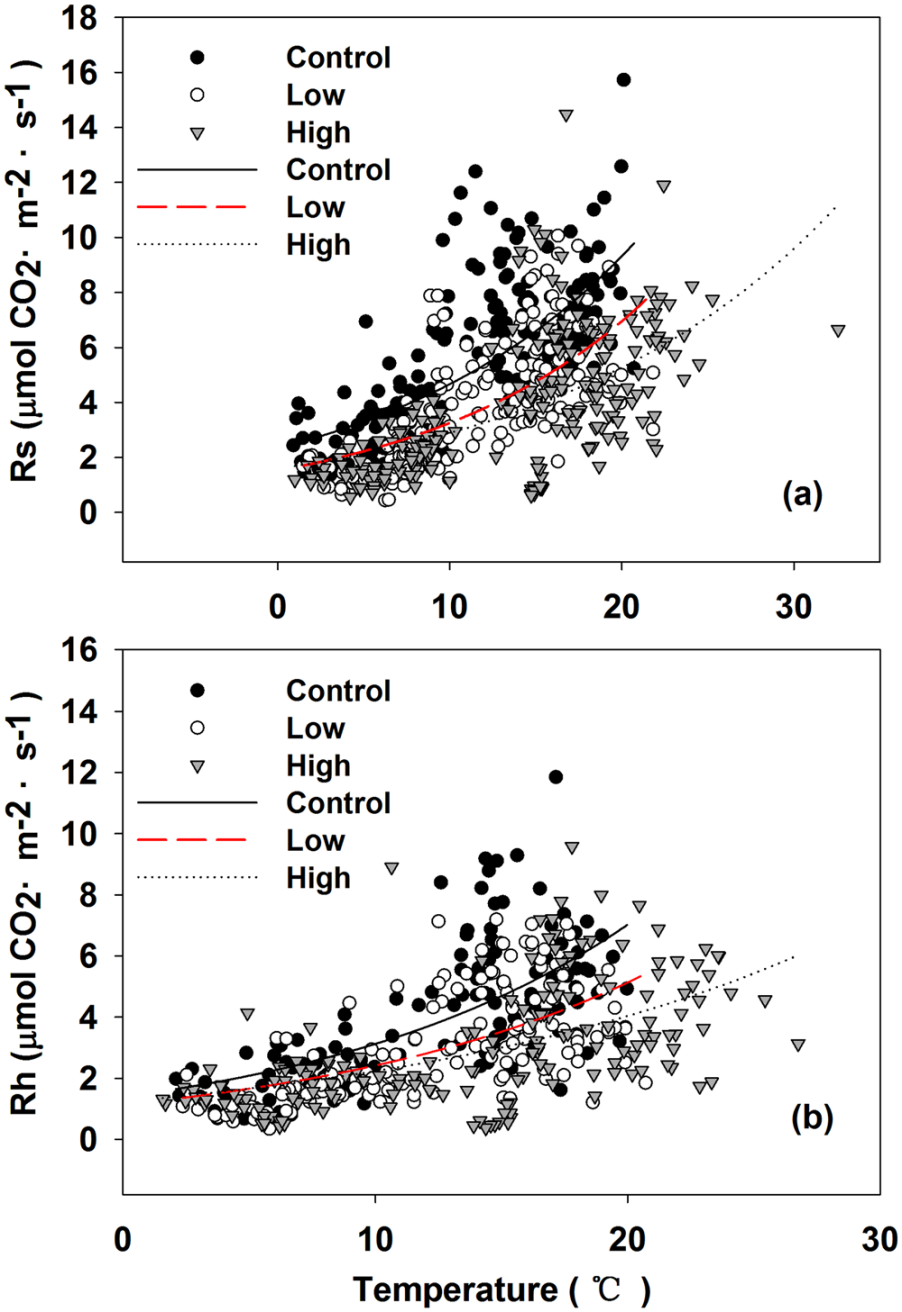
Seasonal variation of total soil respiration rates (*R*s) (**a**), heterotrophic respiration rates (*R*h) (**b**) against soil respiration at a 5 cm depth for the control, low, and high burning severity plots.

Figure 3 and Table 1 illustrated the relationship between the *R*s and *R*h and the soil temperature at the depth of 5 cm and the regression equations in the three types of plots, respectively. Soil respiration increased exponentially with soil temperature. Compared with the control plot, the low burning severity plot had a higher *Q*_10_ after fire disturbance, while the high burning severity plot had a decreased *Q*_10_. The *Q*_10_ in the control and low burning severity plot was 1.4~1.5 times higher than that in the high burning severity plot (Table 1).

### Effects of fire disturbance on annual C efflux

**Figure 4:**
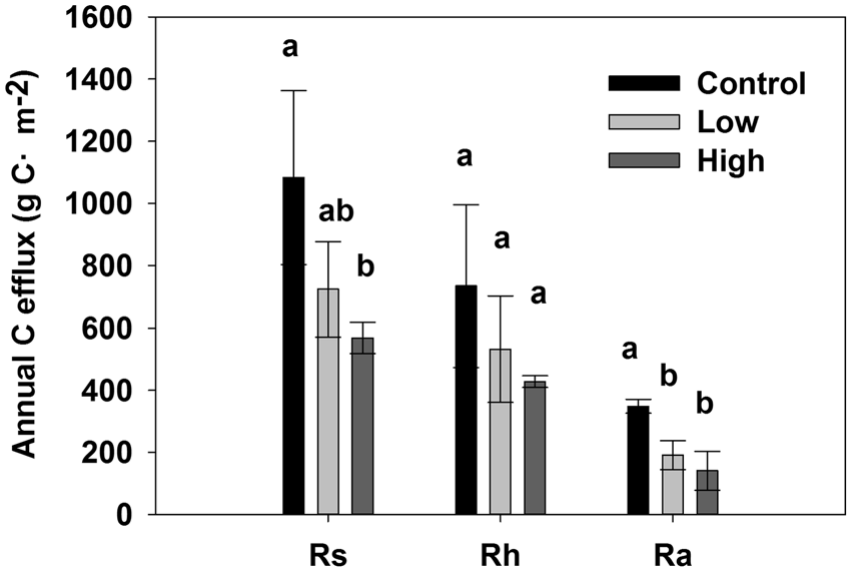
Comparison of the mean annual C efflux in the control, low and high burning severity plots. *R*s, *R*h, and *R*a represent the total soil respiration, heterotrophic and autotrophic respiration, respectively. The error bars represent the standard deviation, and the different lowercase letters are significant at the 95% level.

The mean annual C efflux of *R*h in the control, low and high burning severity plots during the 2010–2012 periods was 735 ± 261, 533 ± 172 and 428 ± 19 g C·m^−2^ (Fig. 4), and was approximately 68%, 74%, and 75% of the mean annual C efflux of *R*s, respectively. The mean annual C efflux of *R*a in the high burning severity plot was significantly lower than that in the control plot (*P* < 0.05, Fig. 4).

## Discussion

Our study first quantified the effects of fire severity on the soil respiration rates and its component change in a Dahurian Larch (*Larix gmelinii*) forest in Northeast China. The *R*s in the Dahurian Larch (*Larix gmelinii*) forest decreased with an increased fire burning severity. The *R*a after high burning severity fires decreased, which decreased the *R*s. Our results indicated that wildfires still affected the *R*a after 5–7 years of fire disturbances. The soil respiration after fire disturbance in the Da Xing’an Mountains was triggered by a higher soil temperature, which can explain a large fraction of soil respiration and its component variability.

The mean *R*s in our study sites (5.29 ± 0.48 μmol CO_2_·m^−2^ s^−1^) was higher than that (2.32–3.88 μmol CO_2_·m^−2^ s^−1^)^28, 29^ in the temperate Dahurian Larch (*Larix gmelinii*) forest and was in the range of the results in other boreal forests (1.14–14.0 μmol CO_2_·m^−2^ s^−1^)^29–32^. Several studies^33–35^ have indicated that the *R*h accounts for approximately 50%–68% of *R*s across different forest ecosystems, which was broadly similar to our study (68~70%).

The reduction of soil respiration by fires depends on the fire severity and duration^36^. Our results confirmed this finding and indicated that the soil respiration decreased with an increased fire severity. The effects of fire disturbance on soil respiration can also be influenced by the proportion of *R*a and *R*h^34, 37^. In our study, the different severity fires had no significant effect on the *R*h. Fire disturbance, however, significantly decreased the *R*a. In the boreal forest, the *R*s was reduced by approximately 53%~67% in the first and second year after the fire disturbance due to removal of the decomposed humic materials^13^. This leads to a reduction in *R*h after a fire disturbance because of the litter and surface organic carbon reduction after the fire disturbance^32^. However, Hicke *et al*.^25^ and Muñoz-Rojas *et al*.^18^ found that the heterotrophic soil respiration rates increased after fire disturbance due to the decomposition of a large amount of combustible substances. Fire removes the vegetation cover and resets the vegetation succession^38^, and a high quality and quantity of detritus increases the decomposition rates by microbes in the early stage after fire disturbance^39^. However, due to a lower net primary productivity during the process of initial system recovery, the *R*h begins to decrease after two years of fire disturbances. The *R*h after five years of fire disturbances decreases to the level before fire disturbance^25^. Although fire may restrain *R*a due to the root mortality, this effect is usually shrouded by the short-term increase of *R*h after a fire disturbance because the *R*h contributes the majority of *R*s^5^. Compared with a low severity fire disturbance, a high severity fire disturbance significantly affects the *R*a, which may result from the more serious damage of larch roots by high severity fires^17, 40^. Burke *et al*.^30^ indicated that soil respiration did not significantly change after fires in Northern Canada but declined after two years of fires and then recovered to the pre-fire level after seven years of fire disturbances. The high burning severity fires still significantly affected the *R*s after five years of fire disturbances. The decrease in *R*s may stem from the reduction in fine root biomass and canopy removal after several years of burning^41^. The loss of *R*a is due to the plant death and the decrease in the soil organic carbon (SOC) quality after several years of fire disturbances^23^.

The annual soil C efflux in this area was consistent with other studies (511 to 1300 g C m^−2^)^42^. The low and high severity fires cause the approximately 33% to 47% decrease in the annual C efflux. The annual soil C efflux in the Dahurian Larch (*Larix gmelinii*) forest of Northeast China decreased with an increased fire burning severity. Wildfires caused uncertainties in the estimation of the annual C efflux after fire disturbance in this area. Therefore, we need to monitor the long-term change in CO_2_ fluxes after fire disturbance. The long-term effects of wildfire on the *R*s depend on fire severity, fire duration and forest management measures, which will directly influence the post-fire vegetation restoration, the recovery of microbial populations and the variability of the quantity and quality of SOC^32, 43^. Fire can cause an imbalance of soil carbon in boreal forest ecosystems. If the frequency and severity of fires continue to increase, the permafrost in boreal forests will degrade and enhance decomposition, which will cause a transition of the boreal forest ecosystems from a net C sink to a source^44^. Our findings indicated that the forest soil after high severity fires continues to be a C source after 5–7 years of fires.

Soil temperature and moisture strongly influence soil respiration changes^45^, and soil temperature is the main factor controlling soil respiration during the growing season^46^. Due to the reciprocal interactions effect between soil temperature and moisture, it is difficult to control and distinguish their effects under field conditions^47^. Our results suggested that the high *R*s is generally triggered by the high temperature during fires. The high soil temperature in high burning severity sites was due to the reduction of vegetation cover after fire and the increasing exposure of the soil surface to solar radiation and the decrease in transpiration, limiting the cooling effect of latent energy in the fire burning area^48^. The variation of soil temperature at a 5-cm depth was consistent with the *R*s. In contrast, the soil moisture did not show a close relationship with *R*s (Fig. 1). The soil moisture did not significantly change during the growing season because of the saturated soil moisture in this area. Recently, a few studies suggested that soil moisture can significantly affect the *R*s only when the soil is in an extreme environment^49^. Although the exponential model of the interaction of soil temperature and moisture was the best-fitted curve to explain the *R*s in the control and high burning severity sites, temperature is still the dominant factor to explain the variation of the *R*s in this area (Table 1).

The *R*s exponentially increased with an increased soil temperature at a 5-cm depth by a *Q*_10_ of 2.36, which is within the range of results from other studies. The *Q*_10_ in Dahurian Larch (*Larix gmelinii*) forest during the growing season ranged from 1.5 to 5.7^50, 51^. Our results showed that a high burning severity decreased the *Q*_10_ after a fire disturbance. The *Q*_10_ in the control and low burning severity sites was 1.4~1.5 times higher than that in the high burning severity sites. According to the recent studies, the *Q*_10_ not only reflects the soil respiration sensitivity to temperature but also expresses the combined response to fluctuations in temperature, root biomass, moisture conditions, and substrate quality^37^. The variation of the *Q*_10_ after fire disturbance may result from the effects of fires on root material because low and moderate severity fires provide more labile carbon in burnt soil versus stable carbon, which will accelerate the root assimilate SOC in the carbon, while high burn severity fires will destroy root structures and cause the loss of the labile fraction of SOC in the atmosphere^52, 53^. The *Q*_10_ of *R*a was higher than that of *R*h in boreal forests^54^, which highlights the importance of the *R*a in regulating the *Q*_10_ of *R*s^37^. The higher root respiration may accelerate the soil organic matter decomposition rate^20^. The decomposition of more recalcitrant soil organic matter may cause the high *Q*_10_^2^. Therefore, further studies are necessary to explore the effect of fires on the *Q*_10_ in root and rhizosphere respiration. This can be used to understand not only the response of soil respiration to temperature but also the mechanisms behind *R*s following fire.

Our finding can provide a scientific basis for the post-fire vegetation restoration in the Daxing’an Mountains. The results of this research further demonstrate that the boreal forest ecosystems of China, particularly in the background of global climate change, are areas sensitive to temperature change. Future efforts are required to fully understand the longer-term variations in soil respiration and its component changes following extreme climate events such as high burning, severe wildfires in Northeast China.

## Methods

### Study area

The research area is located at the Daxing’an Mountains, Nanweng River Forest Ecological Station, Northeast China (51°05′07″N–51°39′24″N, 125°07′55″E–125°50′05″E). The elevation in this area ranges from 500 m to 800 m. The climate is a cold temperate continental monsoon zone. The average annual temperature is −3 °C. There are approximately 2500 annual sunshine hours, and the frost-free period is approximately 90 to 100 days. The annual precipitation is 350 mm to 500 mm. The zonal soil is Podzol. The dominant herb species include *Lespedeza bicolour* Turcz., *Rosa davurica* Pall., *Vaccinium vitis-idaea* L., *Rhododendron Simsii* Planch., *Calamagrostis angustifolia* Kom., and *Maianthemum bifolium*.

### Stand selection and the definition of fire severity

In April 2006, forest fires were caused by lightning in the Songling forest bureau (Nanweng River Forest Ecological Station) of the Daxing’an Mountains, China. The total burned area was approximately 15 × 10^4^ ha. In the fire disturbance area, we classified the fire burning severity according to the depth of the burned organic soil, which is commonly used in boreal forest ecosystems^55–57^. Moreover, we also referred to the consumption of the aboveground biomass, tree mortality, and the bark char height to define the fire severity, which would help us to understand the fire damage to the forest for each burning severity. For the high burning severity, the depth of the burned organic soil was 15.0 ± 1.4 cm; the understory shrubs, litter, and duff layers were completely burned out; the bark char height was 2.5–5.5 m; and the tree mortality was approximately 85%. For the low burning severity, the depth of the burned organic soil was 3.8 ± 0.6 cm, the approximately 25% of the understory shrubs was burned, the bark char height was 1.8–2.4 m, and approximately 20% of the trees died. We selected three replicated plots in each fire severity area to conduct our investigation and selected the nearby unburnt area as the control plots to compare the results with the burned plots. Nine plots (3 plots for high burning severity +3 plots for low burning severity +3 plots as the unburnt control) were selected in our research. The size of each plot was 400 m^2^ (20 m × 20 m), and all plots were established in October 2009.

### Soil respiration measurement

The *R*s was measured by using an Li-8100-103 and Li-8100 portable automatic measuring system for soil carbon flux (Li-Cor, Inc., Lincoln, NE, USA). Five polyvinylchloride (PVC) soil rings (inner diameter 19 cm, height 7 cm) were randomly laid in each plot. Fifteen soil rings (5 soil rings ×3 replicate plots) measuring *R*s were in each fire burning severity plot. The PVC ring remained in the same position throughout the measurement period. The trench method was used to measure the soil heterotrophic respiration (*R*h)^58^. Four 50 cm × 50 cm quadrats were established 2–3 m outside each plot. A trench (45–50 cm depth) was dug in each quadrat, and all roots were removed from the trench severing connections between the plant roots and the trench cross-section. A double-layered plastic cloth was laid in the cross-section of each trench to prevent connection between the trench and any plant roots, and the soil was then replaced. A PVC soil ring was placed in the centre of each quadrat following the same method as above. The CO_2_ flux of trenched quadrat PVC rings was treated as *R*h, including microbial, soil faunal respiration, and CO_2_ emitted by soil organic matter decomposition, while that in non-trenched plots was treated as *R*s. The difference of between *R*s and *R*h was assumed to be *R*a. A total of twelve *R*h soil rings (4 quadrats ×3 replicate plots) was used to measure the *R*h in each fire burning severity. The *R*a account for the ratio of *R*s defined as RC (*R*a/*R*s) was used to represent the relative contribution of root respiration to soil respiration.

The soil respiration rate was measured monthly from May to September in the years 2010–2012. The measurement time lasted approximately two minutes for each soil respiration ring. Each measurement was conducted from 9:00 AM to 11:00 AM for a total of 81 (45 non-trenched soil rings for *R*s +36 trenched soil rings for *R*h) measurements within two days.

### Soil temperature and soil moisture

The soil temperature (*T*) and soil moisture (*W*) were measured by using a temperature probe (Licor p/n8100–201) and soil volumetric water content probe (ECH20 EC-5; p/n 8100-202) at a depth of 5 cm. The measurements of soil temperature and moisture synchronized with the measurement of the soil respiration.

### Statistical analysis

The data were processed and analysed using SPSS 19.0 statistical software (SPSS Institute, Inc., Chicago, IL, USA). Differences in variables between the burning and control plots were tested by analysis of variance (ANOVA), and comparisons between means were performed with the least-significant differences (LSD) test. All statistical analyses were performed with a significance level of 0.05.

### Soil respiration model

The fitting model for the soil respiration rate and soil temperature in the growing season was developed by an exponential model. The goodness-of-fit of the models were quantified using the coefficient of determination (*R* ^2^) and residual analyses. The regression model between soil respiration and soil temperature is shown as Eq. 1 ^59^:

1 $$R{\rm{s}}=\alpha \times {e}^{\beta \times T}$$

where *R*s is the soil respiration (µmol CO_2_·m^−2^ s^−1^), *T* is the soil temperature at a depth of 5 cm (°C), and *α* and *β* are regression coefficients.

At a daily time step, we developed an exponential model that was used to describe the effects of soil temperature and soil moisture on soil respiration. Logarithmic transformation of *R*s was required to achieve linearity and homoscedasticity. The regression model is shown as Eq. 2:

2 $$Ln({R}s)=\alpha +\beta \times T+\varepsilon \times W+\omega \times T\times W$$

where *Ln* (*R*s) is the logarithmic transformation of *R*s that was applied to achieve linearity and homoscedasticity; *T* is soil temperature at −5 cm (°C); *W* is the soil moisture at −5 cm (%); *T* × *W* is the interaction effect of *T* and *W*; and *α*, *β*, *ε*, and *ω* are regression coefficients. A stepwise regression procedure was performed to remove insignificant terms (*P* = 0.05).

### The estimation of the annual C efflux

We measured the soil respiration in the non-growing season from October 2011 to April 2012, which accounted for 11% of the total annual soil respiration in this area. Therefore, we assumed that soil respiration during the non-growing season contributed 11% of the annual C efflux in all plots. The annual C efflux (g C·m^−2^) was estimated by the following equation^20^:

3 $${\rm{Annual}}\,{\rm{C}}\,{\rm{efflux}}=12\times 1800\times {10}^{(-6)}\sum Rs$$

where the figure of 12 is the molecular weight of carbon, and the figure of 1800 is a constant value (unit: second) based on the Campbell Scientific datalogger (Campbell Scientific, Inc., Utah, USA) to record soil temperature and soil moisture every 30 minutes during the period 2010–2012, and *R*s is the soil respiration.

*Q*_10_ is the temperature-sensitive coefficient representing the increase in a process as result of temperature increase at each 10 °C. We used Eqs 1 and 4 to calculate *Q*_10_^28^:

4 $${Q}_{10}={e}^{10\times \beta }$$

where *β* is the regression coefficient calculated from Eq. 1 and *e* is the exponential base.

## References

[1] Bond-lamberty B, Thomson A (2010). Temperature-associated increases in the global soil respiration record. Nature..

[2] Davidson EA, Janssens IA (2006). Temperature sensitivity of soil carbon decomposition and feedbacks to climate change. Nature..

[3] Hashimoto S (2012). A new estimation of global soil greenhouse gas fluxes using a simple data-oriented model. PloS one..

[4] Blankinship JC, Hart SC (2012). Consequences of manipulated snow cover on soil gaseous emission and N retention in the growing season: a meta-analysis. Ecosphere.

[5] Smith DR (2010). Soil surface CO_2_ flux increases with successional time in a fire scar chronosequence of Canadian boreal jack pine forest. Biogeosciences..

[6] Kang H, Fahey TJ, Bae K, Fisk M (2016). Response of forest soil respiration to nutrient addition depends on site fertility. Biogeochemistry..

[7] Vogel JG, Bronson D, Gower ST, Schuur EA (2014). The response of root and microbial respiration to the experimental warming of a boreal black spruce forest. Canadian Journal of Forest Research.

[8] Melillo JM (2002). Soil warming and carbon-cycle feedbacks to the climate system. Science.

[9] Czimczik CI, Trumbore SE, Carbone MS, Winston GC (2006). Changing sources of soil respiration with time since fire in a boreal forest. Global Change Biology.

[10] French NHF, Goovaerts P, Kasischke ES (2004). Uncertainty in estimating carbon emissions from boreal forest fires. Journal of Geophysical Research Atmospheres.

[11] Kasischke ES, Turetsky MR (2006). Recent changes in the fire regime across the north American boreal region—spatial and temporal patterns of burning across Canada and Alaska. Geophysical Research Letters.

[12] Rodríguez A, Durán J, Fernández-Palacios JM, Gallardo A (2009). Wildfire changes the spatial pattern of soil nutrient availability in *Pinus canariensis* forests. Annals of Forest Science.

[13] O’Neill KP, Kasischke ES (2006). Succession-driven changes in soil respiration following fire in black spruce stands of interior Alaska. Biogeochemistry..

[14] Dore S, Fry DL, Stephens SL (2014). Spatial heterogeneity of soil CO_2_ efflux after harvest and prescribed fire in a California mixed conifer forest. Forest Ecology & Management.

[15] Hinzman LD, Fukuda M, Sandberg DV, Chapin FS, Dash D (2003). FROSTFIRE: An experimental approach to predicting the climate feedbacks from the changing boreal fire regime. Journal of Geophysical Research Atmospheres..

[16] Marañón-Jiménez S (2011). Post-fire soil respiration in relation to burnt wood management in a Mediterranean mountain ecosystem. Forest Ecology & Management.

[17] O’Donnell JA, Turetsky MR, Harden JW (2009). Interactive Effects of Fire, Soil Climate, and Moss on CO2 Fluxes in Black Spruce Ecosystems of Interior Alaska. Ecosystems..

[18] Muñoz-Rojas M, Lewandrowski W, Erickson TE, Dixon KW, Merritt DJ (2016). Soil respiration dynamics in fire affected semi-arid ecosystems: Effects of vegetation type and environmental factors. Science of the Total Environment.

[19] Loehman RA, Reinhardt E, Riley KL (2014). Wildland fire emissions, carbon, and climate: Seeing the forest and the trees – A cross-scale assessment of wildfire and carbon dynamics in fire-prone, forested ecosystems. Forest Ecology & Management.

[20] Ma Y (2014). Stand ages regulate the response of soil respiration to temperature in a *Larix principis*-rupprechtii plantation. Agricultural & Forest Meteorology.

[21] Subke JA, Bahn M (2010). On the ‘temperature sensitivity’ of soil respiration: can we use the immeasurable to predict the unknown?. Soil Biology & Biochemistry.

[22] Bahn M (2008). Soil Respiration in European Grasslands in Relation to Climate and Assimilate Supply. Ecosystems..

[23] Uribe C, Inclán R, Sánchez DM, Clavero MA, Fernández AM (2013). Effect of wildfires on soil respiration in three typical Mediterranean forest ecosystems in Madrid, Spain. Plant and Soil.

[24] Widén B, Majdi H (2001). Soil CO_2_ efflux and root respiration at three sites in a mixed pine and spruce forest: seasonal and diurnal variation. Canadian Journal of Forest Research.

[25] Hicke JA (2003). Post-fire response of north American boreal forest net primary productivity analyzed with satellite observations. Global Change Biology.

[26] Xu, H. D X’an *Mountains forests in China* 40–43 (Science Press, 1998).

[27] Wu J, Liu Q, Wang L, Chu GQ, Liu JQ (2016). Vegetation and climate change during the last deglaciation in the Great Khingan mountain, northeastern China. PloS one..

[28] Xu M, Qi Y (2001). Soil-surface CO_2_ efflux and its spatial and temporal variations in a young ponderosa pine plantation in northern California. Global Change Biology.

[29] You W, Wei W, Zhang H, Yan T, Xing Z (2013). Temporal patterns of soil CO_2_ efflux in a temperate Korean Larch (*Larix olgensis* Herry.) plantation, Northeast China. Trees.

[30] Burke RA, Zepp RG, Tarr MA, Miller WL, Stocks BJ (1997). Effect of fire on soil-atmosphere exchange of methane and carbon dioxide in Canadian boreal forest sites. Journal of Geophysical Research Atmospheres.

[31] Rayment MB, Jarvis PG (2000). Temporal and spatial variation of soil CO_2_ efflux in a Canadian boreal forest. Soil Biology & Biochemistry.

[32] O’Neill KP, Kasischke ES, Richter DD (2002). Environmental controls on soil CO_2_ flux following fire in black spruce, white spruce, and aspen stands of interior Alaska. Canadian Journal of Forest Research.

[33] Nakane K, Kohno T, Horikoshi T (1996). Root respiration rate before and just after clear-felling in a mature, deciduous, broad-leaved forest. Ecological Research..

[34] Lin G, Ehleringer JR, Rygiewicz PT, Johnson MG, Tingey DT (1999). Elevated CO_2_ and temperature impacts on different components of soil CO_2_ efflux in Douglas-fir terracosms. Global Change Biology.

[35] Buchmann N (2000). Biotic and abiotic factors controlling soil respiration rates in *Picea abies* stands. Soil Biology & Biochemistry.

[36] Weber MG (1990). Forest soil respiration after cutting and burning in immature aspen ecosystems. Forest Ecology and Management.

[37] Boone RD, Nadelhoffer KJ, Canary JD, Kaye JP (1998). Roots exert a strong influence on the temperature sensitivityof soil respiration. Nature..

[38] Pereira P, Úbeda X, Martin DA (2012). Fire severity effects on ash chemical composition and water-extractable elements. Geoderma..

[39] Rutigliano FA (2007). Impact of fire on fungal abundance and microbial efficiency in C assimilation and mineralisation in a Mediterranean maquis soil. Biology and Fertility of Soils.

[40] Richter, D. D., O’Neill, K. P. & Kasischke, E. S. Post-fire stimulation of microbial decomposition in black spruce (*Picea mariana* L.) forest soils: a hypothesis. *Ecological studies* 197–213 (Springer New York, 2000).

[41] Sullivan BW (2011). Wildfire reduces carbon dioxide efflux and increases methane uptake in ponderosa pine forest soils of the southwestern USA. Biogeochemistry..

[42] Bond-Lamberty B, Wang C, Gower ST (2004). A global relationship between the heterotrophic and autotrophic components of soil respiration?. Global Change Biology.

[43] Pausas JG, Keeley JE (2014). Evolutionary ecology of resprouting and seeding in fire-prone ecosystems. New Phytologist..

[44] Brown DRN (2015). Interactive effects of wildfire and climate on permafrost degradation in Alaskan lowland forests. Journal of Geophysical Research Biogeosciences.

[45] Raich JW, Schlesinger WH (1992). The global carbon dioxide flux in soil respiration and its relationship to vegetation and climate. Tellus..

[46] Savin MC, Görres JH, Neher DA, Amador JA (2001). Biogeophysical factors influencing soil respiration and mineral nitrogen content in an old field soil. Soil Biology & Biochemistry.

[47] Ngao J, Longdoz B, Granier A, Epron D (2007). Estimation of autotrophic and heterotrophic components of soil respiration by trenching is sensitive to corrections for root decomposition and changes in soil water content. Plant and Soil.

[48] Irvine J, Law BE, Hibbard KA (2007). Post-fire carbon pools and fluxes in semiarid ponderosa pine in Central Oregon. Global Change Biology.

[49] Rey (2011). Impact of land degradation on soil respiration in a steppe (*Stipa tenacissima* L.) semi-arid ecosystem in the SE of Spain. Soil Biology & Biochemistry.

[50] Yang J, Wang C (2006). Effects of soil temperature and moisture on soil surface CO_2_ flux of forests in northeastern China. Journal of Plant Ecology.

[51] Wang QF, Wang CK (2008). Vernal soil respiration of *Larix gmelinii* Rupr. forests transplanted from a latitudinal transect. Acta Ecologica Sinica.

[52] Thornley JHM, Cannell MGR (2001). Soil carbon storage response to temperature: an hypothesis. Annals of Botany.

[53] Conant RT (2011). Temperature and soil organic matter decomposition rates – synthesis of current knowledge and a way forward. Global Change Biology.

[54] Lavigne MB (2003). Soil respiration responses to temperature are controlled more by roots than by decomposition in balsam fir ecosystems. Canadian Journal of Forest Research.

[55] Kasischke ES, Hoy EE (2012). Controls on carbon consumption during Alaskan wildland fires. Global Change Biology.

[56] Turetsky MR (2011). Recent acceleration of biomass burning and carbon losses in Alaskan forests and peatlands. Nature Geoscience..

[57] Johnstone JF, Chapin FS (2006). Effects of soil burn severity on post-fire tree recruitment in boreal forest. Ecosystems..

[58] Hanson PJ, Edwards NT, Garten CT, Andrews JA (2000). Separating root and soil microbial contributions to soil respiration: A review of methods and observations. Biogeochemistry..

[59] Lloyd J, Taylor JA (1994). On the temperature dependence of soil respiration. Functional Ecology..

